# Combining expression of GPC3 in tumors and CD16 on NK cells from peripheral blood to identify patients responding to codrituzumab

**DOI:** 10.18632/oncotarget.23830

**Published:** 2018-01-02

**Authors:** Gong Chen, Ya-Chi Chen, Bernhard Reis, Anton Belousov, Lori Jukofsky, Christine Rossin, Axel Muehlig, Chao Xu, Laurent Essioux, Toshihiko Ohtomo, Laura Di Laurenzio, Oscar Puig, Ray Lee

**Affiliations:** ^1^ Roche Pharmaceutical Research and Early Development, Roche Innovation Center New York, New York, NY, USA; ^2^ Roche Pharmaceutical Research and Early Development, Roche Innovation Center Basel, Basel, Switzerland; ^3^ Roche Pharmaceutical Research and Early Development, Roche Innovation Center Penzberg, Penzberg, Germany; ^4^ Chugai Pharmaceutical Co., Ltd., Tokyo, Japan

**Keywords:** hepatocellular carcinoma, antibody-dependent cellular cytotoxicity (ADCC), NK cell, GC33 monoclonal antibody, predictive biomarker

## Abstract

**Background:**

Codrituzumab, a monoclonal antibody targeting an oncofetal protein glypican-3 (GPC3) expressed on cell surface of hepatocellular carcinoma (HCC) induces antibody-dependent cellular cytotoxicity (ADCC) and inhibits tumor growth in preclinical studies. Based on this mechanism, tumor GPC3 expression and CD16 expression on NK cells, which are the effector cells of ADCC, were investigated to correlate with codrituzumab's clinical efficacy in patients with advanced HCC.

**Results:**

Joint analyses of the two biomarkers revealed that both high levels of GPC3 and CD16 were required for patients to benefit from codrituzumab; lack of either one of them would lead to a loss of the therapeutic effect.

**Conclusions:**

These results suggest the combination of tumor GPC3 expression and CD16 expression on NK cells from peripheral blood at baseline as a composite biomarker to select HCC patients for codrituzumab.

**Impact:**

The conclusion warrants a future study in an HCC population with both high GPC3 expression and high levels of CD16 at baseline to establish codrituzumab's therapeutic benefit in HCC.

**Methods:**

Data from a phase II clinical trial of codrituzumab were used for the analyses. GPC3 expression in baseline tumor biopsies was determined by immunohistochemistry (IHC) analysis, and baseline CD16 expression on NK cells were quantified by peripheral blood lymphocyte immunophenotyping. According to high or low expression of GPC3 and CD16, different patient subgroups were formed; for each subgroup, overall survival of patients having high codrituzumab exposure was compared to that of patients receiving placebo.

## INTRODUCTION

Glypican-3 (GPC3) is a heparan sulfate proteoglycan attached to the cell membrane via glycophosphatidylinositol (GPI) anchor [1]. GPC3 is highly expressed in 60–70% of hepatocellular carcinoma (HCC) based on immunohistochemistry (IHC) examination of surgically resected HCC tissues [2–8]. Multiple mechanisms of the role of GPC3 in HCC were reported. GPC3 can stimulate the growth of HCC cells through the canonical Wnt signaling pathway [9], and it also modulates cell proliferation by negatively regulating bone morphogenetic protein 7 (BMP-7) signaling [10]. Expression of GPC3 was shown to be associated with SULF2-mediated cell growth and increased binding of fibroblast growth factor 2 (FGF2), which was supported by the observation that knockdown of GPC3 lessened FGF2 binding in SULF2-expressing HCC cells [11]. GPC3 activates the insulin-like growth factor (IGF)-signaling pathway by binding to IGF2, activating IGF-1R and triggering the phosphorylation of IGF-1R and extracellular signal-regulated kinase (ERK) [12]. Membrane overexpression of GPC3 recruits M2-polarized tumor-associated macrophages (TAM) into human HCC tissues, which may promote HCC progression and metastasis [13].

A recombinant humanized monoclonal antibody, codrituzumab (GC33 or RO5137382), binds to the juxtamembrane domain of GPC3 with high affinity. It was shown to induce antibody-dependent cellular cytotoxicity (ADCC) and tumor growth inhibition in mouse xenograft models, where natural killer (NK) cells can play a major role as effector cells for the ADCC [14]. In contrast, complement-dependent cytotoxicity contributes minimally for codrituzumab's activity [15]. Note that the number of NK cells in peripheral blood and tumor tissues of HCC patients has been shown to be positively correlated with their survival and prognosis; given NK cell's crucial roles in hepatocarcinogenesis, NK cell-based immunotherapies may hold a great promise for HCC, with some attempts being made in on-going human trials [16].

**Figure 1:**
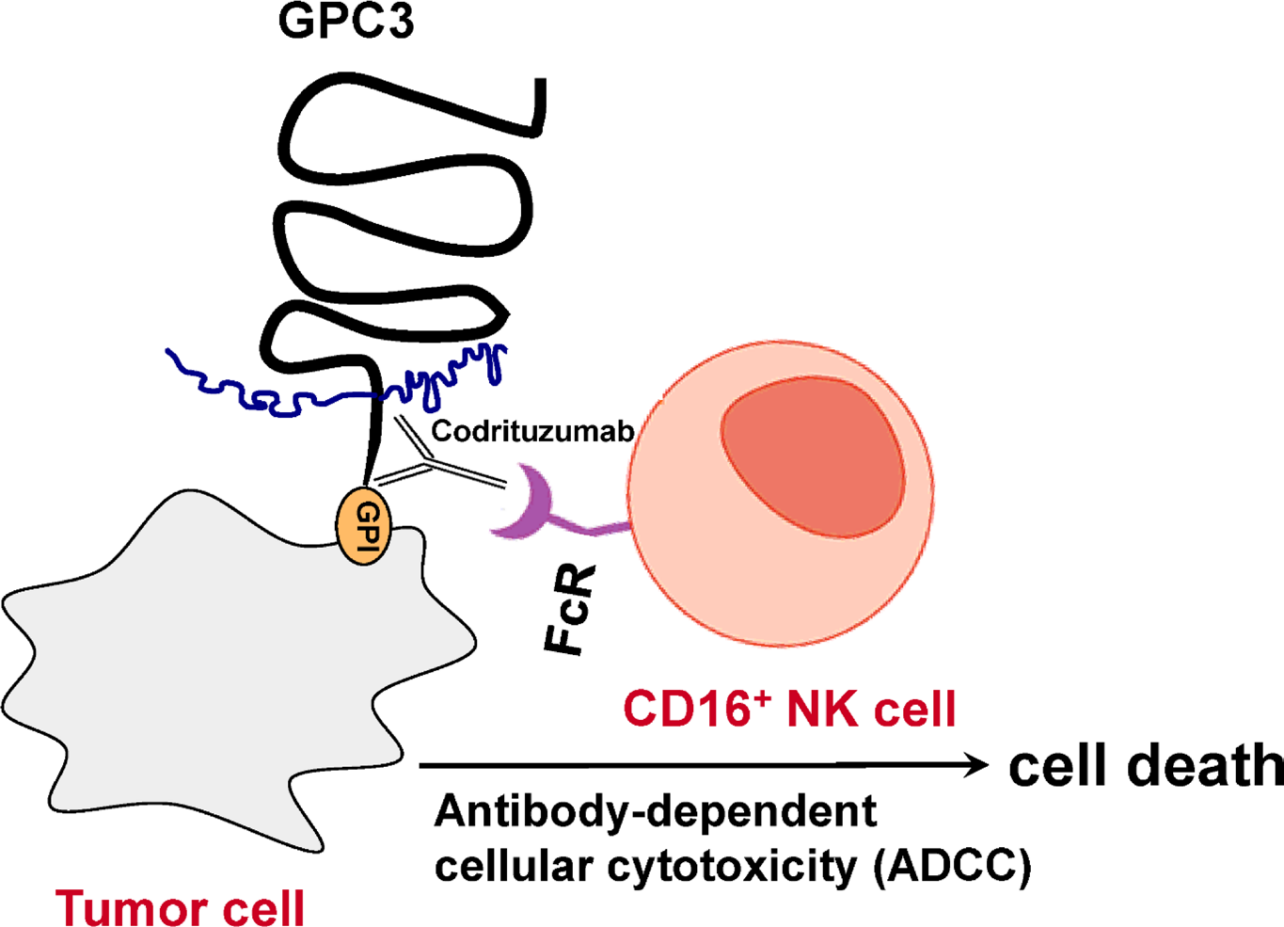
Hypothetic mechanism of action: Antibody-dependent cellular cytotoxicity (ADCC)

In ADCC, an antibody interacts with the cell surface-associated antigens on target cells. The Fc (crystallizable fragment) portion of the antibody recruits the effector cells (predominantly NK cells [17]) through their Fc receptors, which leads to an engagement between the effector cells and target cells to trigger ADCC to induce target cell death [18]. With ADCC as a major mode of action or part of antitumor effects, several anti-cancer therapeutic mAb have been approved and many others are in development [17, 19]. Figure 1 illustrates NK cell-mediated ADCC by codrituzumab under the hypothetic mechanism. When a tumor cell is expressing GPC3 on the surface, codrituzumab binds GPC3 and uses its Fc portion to recruit FcR(Fc receptor)-bearing NK cells to kill the tumor cell. Based on this hypothesis, we predict that the higher the target GPC3 expression, the higher the FcR or CD16 expression or the stronger the affinity between the FcR with the Fc portion of the codrituzumab antibody, the more likelihood the ADCC will occur and the better efficacy of codrituzumab can be achieved. In this study, we examine this theory using data from a randomized phase II clinical study [20] to determine whether the combination of GPC3 expression in tumor and CD16 expression on NK cells in peripheral blood can be used as useful biomarkers to identify responders to codrituzumab versus placebo. Specifically, we made the following contributions:

- We revealed that either the GPC3 expression alone or the CD16 expression alone is not sufficient to establish codrituzumab's therapeutic success.
- We explored different cutoffs of the expression of GPC3 and CD16 in their joint setting and recommended cutoffs that could be used in future trials.

## RESULTS

### Individual biomarker analysis of GPC3 IHC and CD16 MESF against codrituzumab treatment effect based on OS

In [21], we briefly discussed the *individual* effect of two potential biomarkers suggested by the mechanism and indicated their association with the benefit to codrituzumab. In the following, we investigate in detail such association to provide a necessary context for our main focus, the *joint* effect of the two biomarkers on identifying responders to codrituzumab.

For GPC3 expression by IHC, patients were stratified into a GPC3-IHC-high group with GPC3 IHC = 2+, 3+ and a GPC3-low group with GPC3 IHC = 0, 1+. A significant treatment effect was observed in the GPC3-high group (HR = 0.39, *p* = 2.5 × 10^−3^), and there was no efficacy for the GPC3-low group (HR = 0.90, *p* = 4.1 × 10^−1^). Alternatively, we categorized patients with GPC3 IHC = 0 as the GPC3-low group versus the GPC3-high group with GPC3 IHC = 1+, 2+, 3+. The significance of the treatment effect held up in the GPC3-high group (HR = 0.39, *p* = 8.1 × 10^−4^) with codrituzumab-treated patients having a prolonged OS in comparison to patients receiving placebo. Similarly, no obvious efficacy could be observed for the GPC3-low patients (HR = 0.90, *p* = 4.4 × 10^−1^).

Next the effect of CD16 MESF level was examined against patients OS. Two different cutoffs at the 25th percentile, 363,594 MESF, and the 50th percentile (median), 233,595 MESF, were examined. Patients in the CD16-MESF-above-the-25th-percentile group respond better to codrituzumab than placebo in terms of OS (HR = 0.44, *p* = 5.6 × 10^−3^); in contrast, the patients with CD16 MESF below the 25th percentile did not show significantly better response to codrituzumab compared to placebo (HR = 0.76, *p* = 3.1 × 10^−1^). Alternatively, if the median was used to group patients, the same contrast can be observed. In patients with CD16 MESF above the median, codrituzumab-treated patients also showed significantly better OS than those who receiving placebo (HR = 0.33, *p* = 2.6 × 10^−3^); on the other hand, for those with CD16 MESF lower than the median, there was no survival benefit for codrituzumab (HR = 0.82, *p* = 3.1 × 10^−1^). Note that the above CD16 MESF analysis excludes six patients who did not have CD16 MESF measurements. Similarly, these patients will be excluded from the analyses below that involve CD16 MESF.

Although the above results and analyses in [21] suggested that high GPC3 expression in tumor is associated with the codrituzumab's benefit and there is a similar association for high CD16 expression in NK cells, these analyses only independently examined the individual association between a single biomarker, either GPC3 expression or CD16 expression, and codrituzumab's treatment effect and the following key questions remained to be answered: Whether a single biomarker, GPC3 alone or CD16 alone, is sufficient for Codrituzumab's therapeutic success? Should both GPC3 and CD16 be used and what are the reasonable cutoffs to determine their high expression levels for identifying patients who can benefit from Codrituzumab's treatment effect? In the following, we presented our efforts to address these questions.

### Combining GPC3 IHC and CD16 MESF for patient stratification

**Figure 2:**
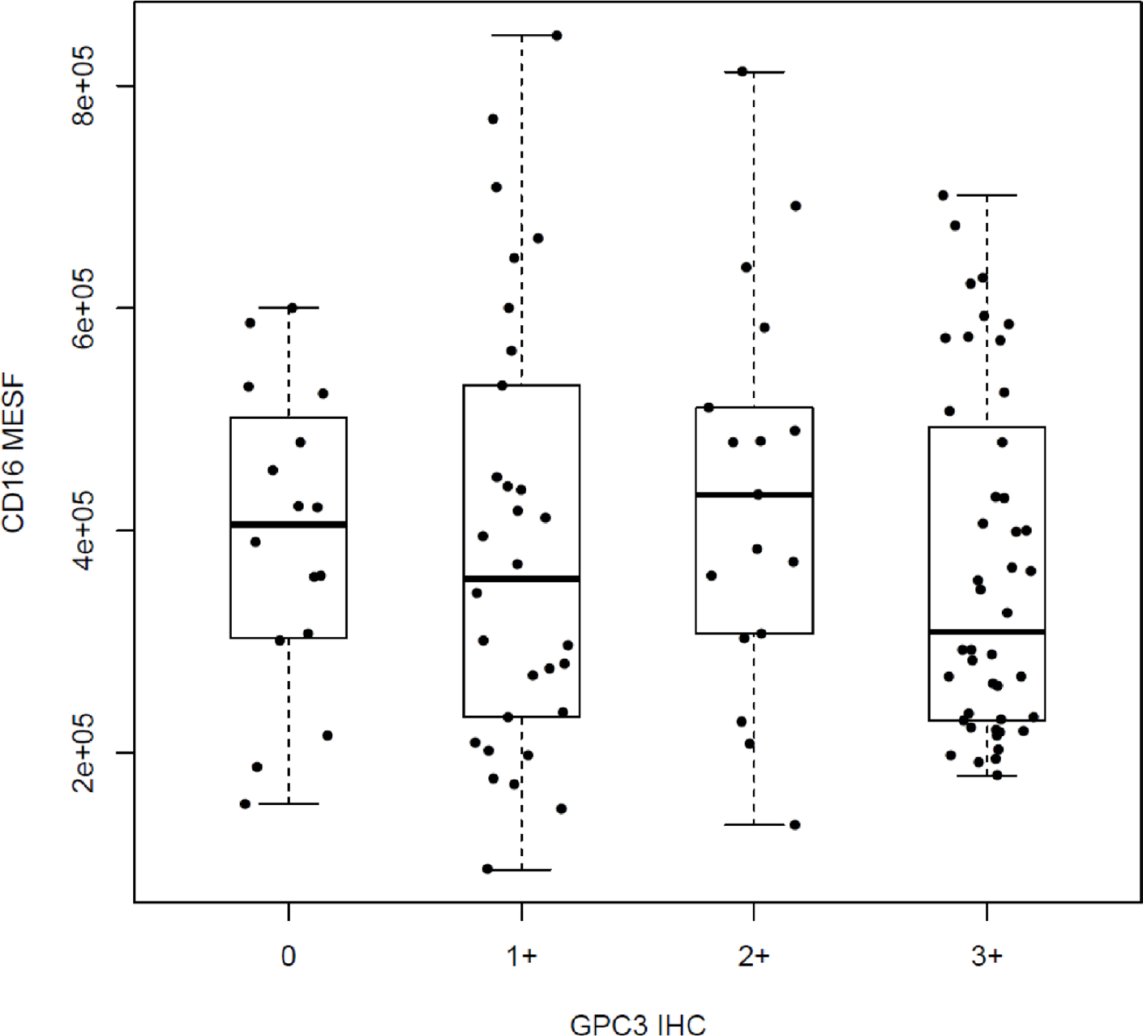
Distributions of CD16 MESF across different GPC3 IHC scores

It was also noted that the distributions of CD16 MESF across the four different GPC3 IHC categories (0, 1+, 2+, and 3+) are not significantly different (*p* = 4.7 × 10^−1^, Figure 2), suggesting that the two biomarkers are independently segregated. Consistently, all pairwise comparisons in CD16 MESF's distributions between any two of the GPC3 IHC categories do not reveal any significant difference in CD16 MESF among the GPC3 IHC categories (all *p* > 0.05). Moreover, patients with high GPC3 IHC do not necessarily have high CD16 MESF, and vice versa. Therefore, GPC3 IHC cannot be used as surrogate of CD16 MESF. In the following, we demonstrated that HR of patients with both high GPC3 IHC and high CD16 MESF is significantly different from HR of patients with high GPC3 IHC and low CD16 MESF. The GPC3 IHC and CD16 MESF must be combined as a composite predictive biomarker predicting codrituzumab's treatment effect.

To examine the association between the joint effect of GPC3 IHC and CD16 MESF at baseline and the codrituzumab treatment effect, we divide the patient population into four groups based on whether a patient's GPC3 IHC is 2+ or higher and whether the CD16 MESF is greater than or equal to the population median. Specifically, the four groups are defined as follows:

- group A with patients having high GPC3 IHC (GPC3 IHC = 2+, 3+) and high CD16 MESF (CD16 MESF ≥ the median);
- group B with patients having high GPC3 IHC and low CD16 MESF (CD16 MESF < the median);
- group C with patients having low GPC3 IHC (GPC IHC = 0, 1+) and high CD16 MESF;
- group D with patients having low GPC3 IHC and low CD16 MESF.

**Figure 3:**
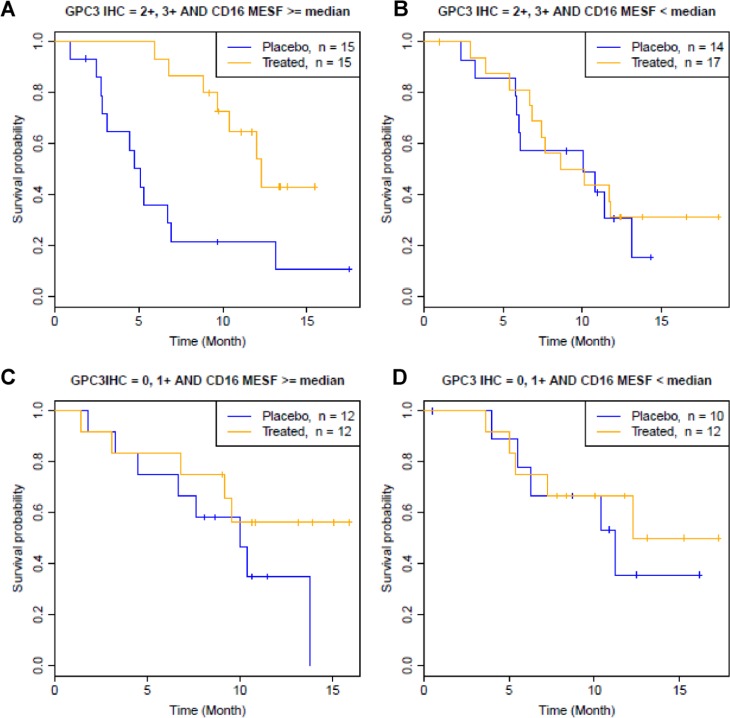
Kaplan-Meier curves by joint GPC3 IHC high/low and CD16 MESF high/low status, where a GPC3-IHC high level is defined by GPC3 IHC = 2+, 3+, and a CD16-MESF high level is defined by CD16 MESF ≥ the median (363,594 MESF) (**A**, **B**) Association between CD16 MESF level and codrituzumab treatment effect given a high level of GPC3 IHC. (**C**, **D**) Association between CD16 MESF level and codrituzumab treatment effect given a low level of GPC3 IHC.

Comparison of group A and group B showed that although both groups have high GPC3 IHC levels, codrituzumab treatment effect is clearly evident in group A with high CD16 MESF levels (HR = 0.14, *p* = 2.6 × 10^−4^, Table 1, Figure 3A) and not in group B with low CD16 MESF levels (*p* = 3.2 × 10^−1^, Table 1, Figure 3B). This result indicated the necessity of high CD16 MESF levels in presence of high GPC3 IHC levels to achieve a robust codrituzumab benefit. On the other hand, in group C, given high levels of CD16 MESF, patients who have low levels of GPC3 IHC did not to respond better to codrituzumab than placebo (*p* = 4.2 × 10^−1^, Table 1, Figure 3C). Comparing to the large treatment effect in group A, the insignificant efficacy in group C suggested that codrituzumab therapeutic effect requires high GPC3 IHC levels. Finally as expected, no difference with respective to OS was seen between codrituzumab and placebo in group D (*p* = 3.7 × 10^−1^, Table 1, Figure 3D), where patients have low GPC3 IHC and CD16 MESF. We thus conclude that both high levels of GPC3 IHC and CD16 MESF are required for patients to benefit from codrituzumab treatment, and lack of either one of them leads to a loss of the treatment effect.

**Table 1:** Hazard ratios and p-values for comparing OS between patients with high codrituzumab exposure and patients receiving placebo in different subgroups defined by joint GPC3 IHC high/low and CD16 MESF high/low status, with a GPC3-IHC-high level defined by GPC3 IHC = 2+, 3+, and a CD16-MESF-high level defined by CD16 MESF ≥ the median (363,594 MESF)

	CD16 MESF >= median	CD16 MESF < median
GPC3 IHC = 2+, 3+	Group A	Group B
	*n* = 30	*n* = 31
	HR (95% CI) = 0.14 (0.05–0.42)	HR (95% CI) = 0.79 (0.30–2.13)
	*p* = 2.6 × 10^−4^	*p* = 3.2 × 10^−1^
GPC3 IHC = 0, 1+	Group C	Group D
	*n* = 24	*n* = 22
	HR (95% CI) = 0.89 (0.26–3.01)	HR (95% CI) = 0.80 (0.21–3.04)
	*p* = 4.2 × 10^−1^	*p* = 3.7 × 10^−1^

Next we examined whether the cutoffs for each parameter could be less stringent to allow a broader patient population to benefit. The cutoff for GPC3 IHC was relaxed to 1+, and the CD16 MESF cutoff relaxed to the 25th percentile. The same conclusion can be made, as shown in Table 2. Specifically, patients whose GPC3 IHC is 1+, 2+ and 3+, and CD16 MESF above the 25th percentile still have a better survival with codrituzumab comparing to those receiving placebo (HR = 0.29, *p* = 7.4 × 10^−4^, Table 2) whereas the treatment effect is not significant for the other 3 groups with low levels in baseline measurements of either biomarkers (HR= 0.69 and 1.31, respectively, Table 2). Selection of this subgroup according to these cutoffs will include the more patients, 67 out of the total 107 (63%), from the study population.

**Table 2:** Hazard ratios and p-values for comparing OS between patients with high codrituzumab exposure and patients receiving placebo in different subgroups defined by joint GPC3 IHC high/low and CD16 MESF high/low status, with a GPC3-IHC-high level defined by GPC3 IHC = 1+, 2+, 3+ and a CD16-MESF-high level defined by CD16 MESF ≥ the 25th percentile (233,595 MESF)

	CD16 MESF >= 25th percentile	CD16 MESF < 25th percentile
GPC3 IHC = 1+, 2+, 3+	Group A	Group B
	*n* = 67	*n* = 24
	HR (95% CI) = 0.29 (0.13–0.62)	HR (95% CI) = 0.69 (0.22–2.17)
	*p* = 7.4 × 10^−4^	*p* = 2.6 × 10^−1^
GPC3 IHC = 0	Group C	Group D
	*n* = 13	*n* = 3
	HR (95% CI) = 1.31 (0.32–5.39)	HR (95% CI): N/A
	*p* = 3.5 × 10^−1^	*p*: N/A

## DISCUSSION

In this study, we reported that, given adequate exposure of codrituzumab, the combination of high GPC3 expression in tumor cells and high CD16 expression on NK cells from peripheral blood at baseline was associated with codrituzumab clinical efficacy in terms of overall survival. The result of our analyses agreed with the potential mechanism of codrituzumab-induced ADCC through recruitment of NK cells, which subsequently induces GPC3-positive tumor cell death. It is also important to know that both factors, relatively high levels of GPC3 IHC and CD16 MESF, may be required for codrituzumab therapeutic benefit. Satisfaction of only one factor might not be sufficient to achieve beneficial treatment effect.

The significant codrituzumab treatment effects estimated under two different sets of cutoffs of GPC3 IHC and CD16 MESF levels further support the robustness of the above conclusion: HR = 0.14 (95% CI: 0.05–0.42) for the population of GPC3 IHC 2+, 3+ and CD16 MESF above the 50th percentile, and similarly, HR = 0.29 (95% CI: 0.13 – 0.62) for the population of GPC3 IHC 1+, 2+, 3+ and CD16 MESF above the 25th percentile. To allow more patients to benefit from codrituzumab treatment, we recommend that future studies to be conducted in a population of GPC3 IHC 1+, 2+, 3+ and CD16 MESF above the 25th percentile, which comprises approximately 63% of total HCC population.

The third component according to the ADCC mechanism of action is the presence of codrituzumab antibody. Therefore, adequate drug exposure of codrituzumab needs to be ensured to show the benefit. The dosing regimen used for patients in the previous trial, 1600 mg every two week after two weekly loadings, was not adequate, as only about 50% of population achieved ~85% or higher target saturation based on the TMDD PK model. As previously discussed, high target saturation is necessary for codrituzumab's therapeutic benefit. The analyses done in this study were therefore based on the group of patients who had a trough level at Cycle 3 Day 1 above 230 μg/mL, the threshold value that is sufficient to achieve desirable target saturation (above 85% target saturation) with codrituzumab.

In conclusion, our biomarker analyses suggest the requirement of tumor GPC3 and blood CD16 as the necessary biomarker to achieve codrituzumab efficacy under the context of adequate codrituzumab exposure. This theory should warrant a future study with a higher dose intensity of codrituzumab in an HCC population with high GPC3 IHC (1+, 2+ and 3+) along with CD16 MESF above the 25th percentile (234K MESF in our established assay) at baseline to establish the therapeutic benefit of codrituzumab in HCC.

## MATERIALS AND METHODS

### Patients

Adult patients with unresectable advanced or metastatic HCC who were previously treated with at least one line of systemic agent and with progressive disease were enrolled in a randomized, placebo-controlled, double-blind, multicenter phase II trial (NCT01507168). Patients received either intravenous (IV) codrituzumab at 1600 mg every two weeks (Q2W) or placebo until disease progression and were followed for overall survival (OS). Please refer to supplementary materials for patient inclusion and exclusion criteria. In summary, 186 patients were randomized in 2:1 ratio to codrituzumab versus placebo. Informed consent was obtained from each patient included in the study and the study protocol conforms to the ethical guidelines of the 1975 Declaration of Helsinki as reflected in a priori approval by the institution's human research committee. Results from this trial showed that codrituzumab could not demonstrate clinical benefit in the all-comer population [21].

A population pharmacokinetic (popPK) covariate model with target-mediated drug disposition (TMDD) was developed using 120 patient's evaluable PK data to project codrituzumab trough concentration of Cycle 3 Day 1 and estimate the target saturation for each individual [22]. The exploratory exposure-response analysis was conducted. A dosing regimen to achieve sustained higher target saturation, such as the median trough level of 230 μg/mL corresponding to 85% target saturation, may be needed for any therapeutic benefit [21]. A longer OS was found to correspond to a higher exposure (with Cycle 3 Day 1 trough concentration ≥ 230 μg/mL) than a low exposure (HR = 0.39, 95% CI: 0.21–0.69). Therefore, the biomarker analyses in this study were focused on patients who have drug exposure ≥ 230 μg/mL, in comparison with those who received placebo. In total, there were 57 patients with high codrituzumab exposure and 56 patients receiving placebo with all the data of baseline demographic variables and important prognostic factors available (Table 3). Distributions of these baseline covariates are not significantly different between the patients with high codrituzumab exposure and the patients receiving placebo (all *p* > 0.05, Table 3).

**Table 3:** Baseline demographics variables and potential prognostic factors

Variable	Description	Placebo	Codrituzumab (high exposure)	*p*
*n*		56	57	
Age	median (range)	62 (36, 79)	62 (31, 80)	0.99
Sex	Male% (Female%)	77% (23%)	75% (25%)	1.00
Race	Asian% (non-Asian%)	45% (55%)	61% (39%)	0.09
Weight	median (range)	68.1 (37.6, 118.1)	69.4 (35.5, 98.1)	0.97
Sum of diameters of measurable target lesions	median (range), log10 scale	1.9 (1.3, 2.5)	1.9 (1.3, 2.4)	0.09
ECOG	Score1% (Score0%)	39 (61)	32 (68)	0.44
Child Pugh	Score 6% (Score5%)	29 (71)	25 (75)	0.67
Macrovascular invasion or extrahepatic spread	Yes% (No%)	79 (21)	79 (21)	1.00
Prior sorafenib treatment	Yes% (No%)	64 (36)	81 (19)	0.06
C-Reactive Protein	median (range)	8.6 (0.0, 141.0)	5.8 (0.0, 160.0)	0.12
Alpha Fetoprotein	median (range), log10 scale	2.4 (0.4, 5.6)	2.3 (0.3, 5.7)	0.66
Alanine Aminotransferase	median (range), log10 scale	1.7 (0.7, 2.2)	1.5 (1.0, 2.0)	0.07

### Determination of GPC3 expression within tumor sites

All the patients enrolled in the study provided a tumor tissue sample to determine the level of GPC3 expression by IHC under central review prior to study entry [20]. Specifically, IHC was performed in fresh tissue or in tissue prepared within 3 months from formalin-fixed, paraffin-embedded (FFPE) blocks of the primary tumor or the metastatic sites. If no archival material was available, a pre-treatment core needle biopsy with minimally 18 gauge needles was obtained. The IHC staining was done on BenchMark XT or ULTRA platforms (Ventana Medical Systems, Inc., Oro Valley, Arizona, see supplementary materials for details). The IHC scoring criteria were specified in Supplementary Table 1. Each patient was assigned a GPC3 IHC score with ordered categorical values 0, 1+, 2+, and 3+, corresponding to increasing levels of GPC3 expression, with scores 0 and 3+ indicating the lowest and highest levels of GPC3 expression, respectively.

### Peripheral blood lymphocyte immunophenotyping and determination of the level of CD16 expressed on NK cells

Whole blood was collected at baseline, and lymphocyte subsets were analyzed using antibody cocktails directed against CD45, CD3, CD4, CD8, CD16, CD19, CD56, CD16/56 and CD335. All antibodies were purchased either from Becton Dickinson or Biolegend. Lymphocyte subsets were quantified by determining relative percentages and absolute counts/μl blood using Trucount tubes (Becton Dickinson, Franklin Lakes, New Jersey). The quantification of CD16 expression level, or fluorescence intensity in units of Molecules of Equivalent Soluble Fluorochrome (MESF), denoted by CD16 MESF, was calculated by converting fluorescence measurements of the NK cell population to an MESF value based on an MESF calibration curve prepared according to fluorescence intensity of calibration beads (Quantum^TM^ MESF bead standard, manufactured by Bang Laboratories, Inc., Fishers, Indianapolis) [23]. The acquisition of samples was performed on FACSCanto^TM^ II instruments equipped with 3 lasers (Becton Dickinson). All flow cytometry testing and analysis of whole blood samples was performed at Covance Central Laboratory Services, Inc.

### Statistical analyses

All statistical analyses were conducted with R 3.0.2 software [24]. Overall survival was used as the response variable, indicating potential clinical benefits. To reduce potential bias caused by baseline demographic variables and prognostic factors in estimating codrituzumab treatment effects for different biomarker-based subgroups, we adjusted all these variables (as listed in Table 3) in Cox proportional hazards regression models––the estimated effects of these baseline variables on OS were taken off from overall HR to estimate the treatment effects. Missing data in adjusted covariates and biomarker variables were excluded from analyses. Regression coefficients, standard errors, and *p*-values were reported in Supplementary Tables 2 and 3. Kaplan-Meier curves were adopted to visualize (marginal) OS comparisons between patients who were treated by codrituzumab and patients who received placebo. Kruskal-Wallis test and Wilcox rank-sum test were used to examine whether the distributions of CD16 MESF are different across different GPC3 IHC categories. Fisher exact test was used to check whether a categorical baseline covariate distributed significantly differently in a placebo group and in a codrituzumab group. Similarly, Wilcox rank-sum test was used to check differential distribution of a continuous baseline covariate in these two groups. Statistical significance was defined as *p* < 0.05.

## SUPPLEMENTARY MATERIALS TABLES


