# Supplementary material for: Combining expression of GPC3 in tumors and CD16 on NK cells from peripheral blood to identify patients responding to codrituzumab
Source: Oncotarget. 2018 Jan 2;9(12):10436–44. doi: 10.18632/oncotarget.23830 (PMC5828203; doi:10.18632/oncotarget.23830)
Supplement: Supplementary file 1 [file oncotarget-09-10436-s001.pdf]

# Combining expression of GPC3 in tumors and CD16 on NK cells from peripheral blood to identify patients responding to codrituzumab

## SUPPLEMENTARY MATERIALS

### Patient inclusion and exclusion criteria

The target population of NP27884 is adult patients (≥18 years) with unresectable advanced or metastatic hepatocellular carcinoma (HCC) previously treated with at least 1 systemic agent, with documented progressive disease, or documented adverse event(s) resulting in discontinuance of that (those) agents.

### Inclusion criteria

- Signed written Institutional Review Board (IRB) /Ethical Committee (EC) approved informed consent form, prior to performing any study-related procedures
- Male or female ≥18 years old
- Life expectancy of at least 12 weeks
- ECOG Performance Status of 0 or 1
- Histologically confirmed unresectable advanced or metastatic hepatocellular carcinoma (without fibrolamellar subtype) patients who are not candidate for or progressed on loco-regional therapy
- Prior treatment with at least 1 systemic agent (such as Sorafenib or other experimental agents), with documented progressive disease after systemic agent(s), or documented adverse event(s) associated with prior systemic agent(s) that resulted in discontinuance of that(those) agent(s)
- Not a candidate for curative treatments (e.g. resection, transplantation)
- Child-Pugh A (score of 5-6)
- Adequate hematologic function:
  - Platelet count ≥ 50 × 109/L
  - Absolute Neutrophil Count ≥ 1,500/µL
  - Hemoglobin ≥ 8.0 g/dL
- Adequate hepatic function:
  - ALT (SGPT) and AST (SGOT) ≤ 5 × ULN
  - Bilirubin ≤ 2 mg/dL
- Adequate renal function:
  - Serum Creatinine ≤ 2 × ULN or calculated Creatinine Clearance ≥ 60 mL/min using Cockcroft and Gault formula
- Ability to provide, for central review, a tumor tissue sample to determine the level of GPC3 expression by IHC. Determination of GPC3 expression level is required prior to study entry.
- Patients must have recovered from effects of any major surgery or significant traumatic injury at least 14 days before the first dose of study treatment
- Measurable disease (by RECIST 1.1) prior to the administration of study treatment
- Negative serum pregnancy test within 10 days prior to commencement of dosing in pre-menopausal women. Women of non-childbearing potential may be included if they are either surgically sterile or have been menopausal for ≥ 1 year
- For women who are not postmenopausal (12 months of amenorrhea) or surgically sterile (absence of ovaries and/or uterus): agreement to use two adequate methods of contraception, including at least one method with a failure rate of < 1% per year (e.g., hormonal implants, combined oral contraceptives, vasectomized partner), during the treatment period and for at least 3 months after the last dose of study drug
- For men: agreement to use a barrier method of contraception during the treatment period and for at least 40 days after the last dose of study drug

### Exclusion criteria

- Child-Pugh B or C
- Known HCC with fibro-lamellar histology
- Known Brain or leptomeningeal metastases
- Patients with a previous malignancy within the past 5 years are excluded (patients who had curatively treated basal cell carcinoma of the skin, early gastrointestinal cancer by endoscopic resection, and/or *in situ* carcinoma of the cervix and other malignancies which were considered cured and deemed by the PI to have no impact on the PFS and OS are allowed after discussion with the study medical monitor)
- Active infectious diseases requiring treatment except for hepatitis B and C
- NCI CTCAE version 4.0 grade 3 hemorrhage within 4 weeks of starting the study treatment
- History of organ allograft including liver transplant

• Anticipated or ongoing administration of anti-cancer therapies other than those administered in this study

• Patients who have had any anticancer treatment within 2 weeks prior to entering the study

• Patients who have not fully recovered from toxicities associated with previous HCC loco-regional or systemic therapies

• Patients receiving Interferon therapy

• Patients with baseline QTc > 470 ms, or patients with baseline resting bradycardia < 45 beats per minutes

• Patients who received anticoagulation or thrombolytic agents for therapeutic purposes (except for low-dose administration for catheter clearance or for prophylactic purposes) within 2 weeks prior to Day 1

• Pregnant or lactating women

• Known HIV positivity or AIDS-related illness

• History of hypersensitivity to similar agents (monoclonal antibody, protein-included drugs, Chinese hamster ovary products)

• Patients with significant concomitant disease determined by the investigator to be potentially aggravated by the investigational drug

• Any psychological, familial, sociological or geographical condition potentially hampering compliance with the study protocol requirements and/or follow-up procedures; those conditions should be discussed with the patient before trial entry.

## GPC3 IHC staining method

Anti-Glypican-3 mouse monoclonal primary antibody (Ventana Medical Systems, Inc or VMSI, catalog number 790–4664) was used together with the ultraView detection kit (VMSI, catalog number 760–500). Horseradish peroxidase (HRP)-conjugated multimer secondary antibody was applied after primary antibody incubation. The HRP conjugate was visualized with hydrogen peroxide in the presence of DAB and copper sulfate. The conjugate, all chromogen reagents, and secondary antibodies were applied using BenchMark XT (VSMI, catalog number 750–700) or ULTRA (VSMI, catalog number 750–600) staining platforms and instrument default times.

**Supplementary Table 1: GPC3 IHC scoring criteria**

| Score | Description |
|---|---|
| 0 | • Absence of membrane staining<br>• Cytoplasmic staining of any intensity in < 10% of tumor cells |
| 1+ | • Membrane staining of any intensity in < 10% of tumor cells<br>**and/or**<br>• Cytoplasmic staining of any intensity > 10% of tumor cells (note that strong cytoplasmic staining, if present, must be in < 50% of tumor cells) |
| 2+ | • Presence of weak to moderate membrane staining in ≥ 10% of tumor cells (note that strong membrane staining, if present, must be in < 10% of tumor cells)<br>**with or without**<br>• Cytoplasmic staining of any intensity >10% of tumor cells (note that strong cytoplasmic staining, if present, must be in < 50% of tumor cells) |
| 3+ | • Strong membrane staining in > 10% of tumor cells with or without cytoplasmic staining<br>**or**<br>• Strong cytoplasmic staining in ≥ 50% of tumor cells |

**Supplementary Table 2: Cox proportional hazards regression coefficients for CD16 MESF or GPC3 IHC as individual biomarkers.** (**A**) A CD16-MESF-high level is defined by CD16 MESF ≥ the median (363,594 MESF). (**B**) A CD16-MESF-high level is defined by CD16 MESF ≥ the 25th percentile (233,595 MESF). (**C**) A GPC3-IHC-high level is defined by GPC3 IHC = 2+, 3+. (**D**) A GPC3-IHC-high level is defined by GPC3 IHC = 1+, 2+, 3+. See Supplementary_Table_2

**Supplementary Table 3: Cox proportional hazards regression coefficients for the combination of CD16 MESF and GPC3 IHC as a biomarker.** (**A**) A CD16-MESF-high level is defined by CD16 MESF ≥ the median (363,594 MESF), and a GPC3-IHC-high level is defined by GPC3 IHC = 2+, 3+. (**B**) A CD16-MESF-high level is defined by CD16 MESF ≥ the 25th percentile (233,595 MESF), and a GPC3-IHC-high level is defined by GPC3 IHC = 1+, 2+, 3+

**A**

| variable | Coef | SE | *p* |
|---|---|---|---|
| Age | –0.02 | 0.02 | $2.3 \times 10^{-1}$ |
| Sex=Male (vs Female) | 0.50 | 0.41 | $2.2 \times 10^{-1}$ |
| Race=ASIAN (vs non ASIAN) | –0.20 | 0.37 | $5.8 \times 10^{-1}$ |
| Baseline_Weight | 0.00 | 0.01 | $8.1 \times 10^{-1}$ |
| Sum_diameter_measurable_target_lesions | 0.44 | 0.59 | $4.6 \times 10^{-1}$ |
| ECOG_Performance_Status_at_Baseline | 0.98 | 0.32 | $2.0 \times 10^{-3}$ |
| Child_Pugh_Score=6 (vs 5) | 0.22 | 0.34 | $5.1 \times 10^{-1}$ |
| Macrovascual_Invasion_or_Extrahepatic=YES (vs NO) | 0.01 | 0.43 | $9.8 \times 10^{-1}$ |
| Prior_Sorafenib_Treatment=YES (vs NO) | –0.71 | 0.31 | $2.1 \times 10^{-2}$ |
| C_Reactive_Protein | 0.00 | 0.01 | $7.2 \times 10^{-1}$ |
| Alpha_Fetoprotein | 0.25 | 0.11 | $3.1 \times 10^{-2}$ |
| Alanine_Aminotransferase | –1.02 | 0.53 | $5.2 \times 10^{-2}$ |
| Treated (vs Placebo) | –1.97 | 0.57 | $5.2 \times 10^{-4}$ |
| GPC3 IHC low AND CD16 MESF high (vs GPC3 IHC high AND CD16 MESF high) | –1.02 | 0.57 | $7.5 \times 10^{-2}$ |
| GPC3 IHC high AND CD16 MESF low (vs GPC3 IHC high AND CD16 MESF high) | –0.75 | 0.52 | $1.5 \times 10^{-1}$ |
| GPC3 IHC low AND CD16 MESF low (vs GPC3 IHC high AND CD16 MESF high) | –0.48 | 0.67 | $4.8 \times 10^{-1}$ |
| Treated x GPC3 IHC low AND CD16 MESF high | 1.85 | 0.88 | $3.6 \times 10^{-2}$ |
| Treated x GPC3 IHC high AND CD16 MESF low | 1.74 | 0.77 | $2.3 \times 10^{-2}$ |
| Treated x GPC3 IHC low AND CD16 MESF low | 1.75 | 0.87 | $4.6 \times 10^{-2}$ |

**B**

| variable | Coef | SE | *p* |
|---|---|---|---|
| Age | –0.02 | 0.02 | $1.9 \times 10^{-1}$ |
| Sex=Male (vs Female) | 0.39 | 0.41 | $3.4 \times 10^{-1}$ |
| Race=ASIAN (vs non ASIAN) | 0.14 | 0.36 | $7.0 \times 10^{-1}$ |
| Baseline_Weight | 0.01 | 0.01 | $6.5 \times 10^{-1}$ |
| Sum_diameter_measurable_target_lesions | 0.33 | 0.58 | $5.7 \times 10^{-1}$ |
| ECOG_Performance_Status_at_Baseline | 0.75 | 0.31 | $1.5 \times 10^{-2}$ |
| Child_Pugh_Score=6 (vs 5) | 0.35 | 0.34 | $3.0 \times 10^{-1}$ |
| Macrovascual_Invasion_or_Extrahepatic=YES (vs NO) | 0.12 | 0.44 | $7.9 \times 10^{-1}$ |
| Prior_Sorafenib_Treatment=YES (vs NO) | –0.60 | 0.31 | $5.7 \times 10^{-2}$ |
| C_Reactive_Protein | 0.00 | 0.01 | $5.5 \times 10^{-1}$ |
| Alpha_Fetoprotein | 0.27 | 0.10 | $4.9 \times 10^{-3}$ |
| Alanine_Aminotransferase | –1.23 | 0.51 | $1.5 \times 10^{-2}$ |
| Treated (vs Placebo) | –1.25 | 0.39 | $1.5 \times 10^{-3}$ |
| GPC3 IHC low AND CD16 MESF high (vs GPC3 IHC high AND CD16 MESF high) | 0.53 | 0.60 | $3.8 \times 10^{-1}$ |
| GPC3 IHC high AND CD16 MESF low (vs GPC3 IHC high AND CD16 MESF high) | –0.29 | 0.48 | $5.5 \times 10^{-1}$ |
| Treated x GPC3 IHC low AND CD16 MESF high | 1.52 | 0.85 | $7.3 \times 10^{-2}$ |
| Treated x GPC3 IHC high AND CD16 MESF low | 0.87 | 0.71 | $2.2 \times 10^{-1}$ |
